# Association between obesity, physical activity, and cognitive decline in Chinese middle and old-aged adults: a mediation analysis

**DOI:** 10.1186/s12877-024-04664-4

**Published:** 2024-01-11

**Authors:** Xin Xu, Yi Xu, Ruolin Shi

**Affiliations:** 1 Operating Room, Department of Nursing, The First Affiliated Hospital of Wenzhou Medical University, Wenzhou, Zhejiang 325000 China; 2 Xinhua Hospital, Shanghai Jiao Tong University School of Medicine, Shanghai, China; 3 Wenzhou Medical University, Wenzhou, Zhejiang China

**Keywords:** Obesity, Physical activity, Cognitive decline, CHARLS, Mediation analysis

## Abstract

**Background and objectives:**

Epidemiological evidence on obesity and cognitive decline in middle and old-aged individuals is controversial and the effect of physical activity in this chain is sparse and limited. This study aimed to characterize the association between obesity and cognitive decline and the mediating role of physical activity.

**Methods:**

Data from China Health and Retirement Longitudinal Study (CHARLS) were used, including 7,392 participants aged ≥ 45 years between 2011 and 2018. Cognitive function was assessed via episodic memory and mental status. The total score of cognitive function was the sum of the above two dimensions (0–31 points). The Group-based trajectory modeling (GBTM) was applied to identify the potential heterogeneity of longitudinal changes in cognitive function. Multivariable ordinal logistic regression was used to investigate associations between obesity and cognitive trajectories, taking body mass index (BMI) as the indicator of obesity. Mediation analysis was performed to examine the potential causal chain in which physical activity mediates the relationship between BMI and cognitive decline.

**Results:**

Of the 7,392 analyzed patients (mean [SD] age, 58.0 [8.5] years; 3,916 [53%] male), the median (interquartile range [IQR]) of BMI was 23.4 (21.1–26.0). Four trajectories were identified by the GBTM model, including the high stable (14.9%), the middle stable (46.0%), the middle decline (29.9%), and the low decline groups (9.2%). After controlling potential confounders, obesity was associated with the low decline groups compared with normal weight (adjusted OR 0.81; 95% CI, 0.70–0.94). Mediation analyses showed that only vigorous physical activity significantly explained 5.94% (95% CI, 0.29-11.60%) of the relationship between obesity and cognitive decline. Sensitivity analyses in different subgroups showed comparable results.

**Conclusion:**

This study suggests that vigorous physical activity mediates less than 10% of the association between obesity and cognitive decline in middle and old-aged adults. Further studies are warranted to explore the potential factors related to the obesity paradox in the cognitive field.

**Supplementary Information:**

The online version contains supplementary material available at 10.1186/s12877-024-04664-4.

## Background

Cognitive decline is a major public health challenge that may lead to mild cognitive impairment or dementia globally [1]. Dementia has become the fifth leading cause of death worldwide, posing a significant burden on long-term care for families and society [2]. The World Health Organization (WHO) estimates that 55 million older adults are living with dementia worldwide, with 15 million residing in China [3]. Furthermore, this number is expected to rise to 30 million by 2050 [4].

Meanwhile, the prevalence of obesity has significantly increased over the past few decades and obesity have been verified to be related to all-cause mortality risk and to worse health conditions [5, 6], while the impact on cognitive impairment or dementia risk remains debated [7]. Previous studies have shown that obesity is detrimental to cognitive impairment and dementia in midlife, accounting for one third of all dementia cases worldwide [8, 9]. Conversely, studies have shown that overweight and obesity may be helpful in late life [9], with a growing number of meta-analyses demonstrating protective effects of body mass index (BMI) on cognitive decline in both midlife and late-life [8, 10], supporting the concept of an “obesity paradox” [11, 12].

Nevertheless, the mechanisms responsible for this paradox remain unclear. One plausible explanation postulates that physical activity has been proposed as a possible mediator in the relationship between obesity and cognitive decline. Recent evidence suggests that individuals with higher BMI may engage in physical activity to mitigate the detrimental effects of their weight on brain health [13]. The favorable impact of physical activity on cognitive function is widely acknowledged, especially in improving overall cognitive function as an important neuroprotective modulator [14]. Nevertheless, the idea still needs to be further proved that the extent to which physical activity mediates the association between obesity and cognitive function.

In this study, we hypothesized that the relationship between obesity and cognitive decline is mediated by physical activity. Based on data from China Health and Retirement Longitudinal Study (CHARLS), we investigated to what extent, if any, physical activity mediated the relationship between obesity and cognitive decline among middle and old-aged Chinese adults and assessed the mediation effect in key important subgroups and on varying degrees of physical activity.

## Methods

### Study design and participants

The data for this study were derived from the China Health and Retirement Longitudinal Study, which is a nationally representative longitudinal cohort study. The study design and patient enrollment have been described previously [15]. Briefly, the study recruited 17,708 participants from 150 counties or districts and 450 villages within 28 provinces in China between June 2011 and March 2012, using the multistage stratified probability-proportional-to-size sampling technique. These respondents were followed up once every 2 years to repeat the survey, and four national waves of data are available to date (waves in 2011, 2013, 2015, and 2018). All participants underwent a standardized questionnaire assessment via one-to-one interviews to collect data on demographic backgrounds, health status and functioning, social and economic status, and retirement information. The overall response rate to the CHARLS was 80.5% at baseline [16]. We excluded 648 individuals younger than 45 years, 5,062 without cognitive function assessments at baseline, 2,740 without cognitive function assessments at 3 follow-up visits, and 1,866 without BMI at baseline. Finally, a total of 7,392 participants were included in the analysis, and 5,582 (75.5%) of them provided blood samples at baseline.

### Cognitive function assessments

The cognitive assessment included two dimensions, episodic memory, and mental status [17]. Episodic memory was assessed by immediate word recall (0–10 points) and delayed word recall (0–10 points). Mental status was assessed using questions from the Telephone Interview of Cognitive Status (TICS) battery, which included items such as orientation, visuo-construction, and attention. Orientation (0–5 points) was assessed by asking respondents to name today’s date (month, day, year, and season) and the day of the week; visuo-construction (0–1 points) was evaluated by testing the ability to redraw a previously displayed figure; attention (0–5 points) was assessed by serial subtraction of 7 from 100 for five times. The total score of cognitive function was the sum of these two dimensions, ranging from 0 to 31. A higher score indicates better cognitive function.

To explore the heterogeneity of cognitive function trajectories, group-based trajectory modeling (GBTM) was employed using the longitudinal data. GBTM is a potential class growth model that identifies distinct trajectory groups and estimates the shape of their cognitive function trajectories over time [18]. To determine the optimal number of trajectory groups and their structure, an iterative process of model selection was conducted. This process involved considering multiple factors, including statistical measures such as *p*-values of model parameters and confidence intervals of trajectory estimates, visual inspection of predicted trajectories, Akike Information Criterion (AIC), Bayesian Information Criterion (BIC), and the average posterior probabilities (AvePP) [19].

### Body mass index

The weight and height were collected at baseline as well as during follow-up interviews. BMI was calculated as weight (kg) divided by height square (m^2^). Refer to the Chinese criteria for adults, underweight was defined as a BMI of less than 18.5 kg/m^2^, normal weight was defined as a BMI between 18.5 and 23.9 kg/m^2^, overweight was defined as a BMI between 24 and 27.9 kg/m^2^, and obesity was defined as a BMI of 28 kg/m^2^ or higher.

### Physical activity

To evaluate the physical activity of the participants, an abbreviated version of the International Physical Activity Questionnaire (IPAQ) was used. This questionnaire is widely recognized as a tool for measuring and soliciting physical activity. Healthy physical activity was defined as at least three times per week and 30 min each time vigorous (i.e., heavy lifting, cycling with a heavy load, and aerobics) or moderate physical activities (such as bicycling at a regular pace, doing tai-chi, walking at a quick pace, etc.). Mild activity intensity was defined as walking.

### Covariates

The study utilized a structured questionnaire to gather baseline data on sociodemographic status and health-related factors. Trained interviewers collected information on age, gender, living residence, marital status, and educational level. Marital status was categorized as either married or another marital status (never married, separated, divorced, and widowed). Educational level was classified into four categories, including no formal education, primary school, middle or high school, and college or above. Additionally, health-related factors were assessed, including self-reported smoking and drinking status (never, former, or current), self-reported physician-diagnosed medical conditions (cardiovascular disease (CVD), diabetes, dyslipidemia, hypertension, and chronic kidney disease), and use of medications for CVD, diabetes, hypertension, and dyslipidemia. A sub-cohort of 5,582 participants underwent metabolic examinations, which included measuring total cholesterol, triglycerides, high-density lipoprotein cholesterol, low-density lipoprotein, fasting plasma glucose, and high-sensitivity C-reactive protein. The study also calculated the estimated glomerular filtration rate using the Chronic Kidney Disease Epidemiology Collaboration’s 2009 creatinine equation. Diabetes was then defined as fasting plasma glucose ≥ 126 mg/dL, current use of antidiabetic medication, or self-reported history of diabetes; dyslipidemia was defined as total cholesterol ≥ 240 mg/dL, triglycerides ≥ 150 mg/dL, low-density lipoprotein cholesterol ≥ 160 mg/dL, high-density lipoprotein cholesterol < 40 mg/dL, current use of lipid-lowering medication, or self-reported history of dyslipidemia; hypertension was defined as systolic blood pressure ≥ 140 mm Hg, diastolic blood pressure ≥ 90 mm Hg, current use of the antihypertensive medication, or self-reported history of hypertension; and chronic kidney disease was defined as estimated glomerular filtration rate < 60 mL/min/1.73 m^2^ or self-reported history of chronic kidney disease.

### Statistical analysis

Normally distributed continuous variables were reported as the means and SDs and nonnormally distributed continuous variables were reported as medians and interquartile ranges (IQRs). Categorical variables were presented as frequencies and percentages. Baseline characteristics are summarized according to BMI categories and compared using the analysis of variance (ANOVA), Mann-Whitney U test, or χ^2^ test, as appropriate.

Associations of BMI and physical activity with 4 cognitive function trajectories were measured by odds ratios (ORs) and 95% CIs based on multivariable ordinal logistic regression models. The BMI was first treated as a categorical variable with normal weight category as the reference group and then treated as a continuous variable with increments of 1 standard deviation (BMI _per SD_) in the ordinal logistic models. We also explored the association between the lipid biomarkers and cognitive trajectory in the sub-cohort underwent metabolic examinations.

**Fig. 1:**
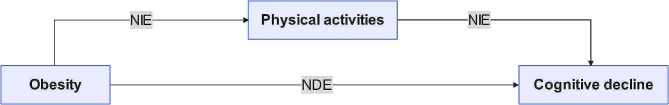
Illustration of mediation effect Abbreviation: NDE, natural direct effect. NIE, natural indirect effect. Total effect = NDE + NIE

To explicate the association of BMI with cognitive decline, indirect associations acting through physical activity as a mediating variable and direct associations not mediated by physical activity were quantified (Fig. 1). A causal mediation analysis under a counterfactual framework was performed, which decomposes the total effect (TE) into two components: the natural direct effect (NDE) and the natural indirect effect (NIE) [20, 21]. The NDE represents the effect of obesity on the cognitive decline that is independent of physical activity, while the NIE represents the effect of obesity on cognitive decline that is mediated by changes in physical activity. The mediation effect is measured by the percentage mediated (PM), which is the percentage of the total effect that is mediated by the mediator [22]. Figure S1 showed the trajectories of cognitive function and its five measurement dimensions stratified by 4 latent trajectory groups, and the high stable and middle stable trajectory groups were clearly distinguished from the middle decline and low decline groups. Therefore, we divided the 4 trajectory groups into a binary outcome (taking high stable and middle stable trajectories as stable groups, middle decline and low decline trajectories as decline groups), to improve the interpretability of the mediation results. Two models were then fitted: a multivariate logistic regression model for physical activity (mediator) conditional on BMI _per SD_ (exposure) and all study confounders and another multivariate logistic regression model for cognitive decline (outcome, decline groups vs. stable groups) conditional on BMI _per SD_, physical activity, and all study confounders. Factors known to be associated with physical activity and cognitive decline were included in the analyses as confounders, which included age, gender, residence, marital status, educational level, smoking status, drinking status, medical history (CVD, diabetes, hypertension, dyslipidemia, and chronic kidney disease), and history of medication use (CVD medications, hypertension medications, diabetes medications, and lipid-lowering therapy). Subgroup analyses were applied to test the robustness of the findings.

All statistical analyses were performed using R 4.2.0 (R core team, Wien, Austria), with two-sided *p*-values < 0.05 used to determine statistical significance.

## Results

### Descriptive characteristics

Table 1 displayed the characteristics of these patients at baseline (2011) categorized by BMI. Of the 7,392 patients included in the analysis, the mean [SD] age was 58.0 [8.5] years old, with 53% [*n* = 3,916] being male. A majority of the patients were married (86% [*n* = 6,367]) and resided in rural areas (60%, [*n* = 4,462]). The most prevalent comorbidity among the patients was hypertension (23% [*n* = 1,867]), followed by CVD (13% [*n* = 1,018]). The majority of participants fell in the normal BMI (40.6% [*n* = 2,999]), followed by the obese (33.2% [*n* = 2,453]) and overweight (21.0% [*n* = 1,556]) categories. Only a small proportion of patients were classified as underweight (5.2%). The average follow-up time of this study was 5.41 years.

**Table 1:** Baseline characteristics of 7,392 participants by body mass index category

Characteristic	Underweight (*n* = 384)	Normal weight (*n* = 2,999)	Overweight (*n* = 1,556)	Obese (*n* = 2,453)	*P* Value ^a^
Age, mean (SD), y	62.7 ± 9.5	58.4 ± 8.6	57.1 ± 8.1	56.3 ± 7.9	< 0.001
Male	217(56.5)	2275(59.3)	1065(47.3)	359(39.2)	< 0.001
Rural residence	273(71.1)	2505(65.3)	1231(54.6)	453(49.5)	< 0.001
Married	319(83.1)	3255(84.8)	1976(87.7)	817(89.2)	< 0.001
Educational level					< 0.001
No formal education	81(21.1)	613(16.0)	334(14.8)	137(15.0)	
Primary school	195(50.8)	1796(46.8)	920(40.8)	370(40.4)	
Middle or high school	107(27.9)	1371(35.7)	957(42.5)	394(43.0)	
College or above	1(0.3)	59(1.5)	42(1.9)	15(1.6)	
Smoking status ^b^					< 0.001
Never	178(46.4)	1977(51.5)	1424(63.2)	640(69.9)	
Former	39(10.2)	343(8.9)	244(10.8)	95(10.4)	
Current	167(43.5)	1518(39.6)	585(26.0)	181(19.8)	
Drinking status					< 0.001
Never	212(55.2)	1979(51.5)	1331(59.1)	584(63.8)	
Former	36(9.4)	322(8.4)	168(7.5)	80(8.7)	
Current	136(35.4)	1538(40.1)	754(33.5)	252(27.5)	
History of comorbidities					
CVD	39(10.2)	438(11.4)	347(15.4)	194(21.2)	< 0.001
Diabetes ^b^	8(2.1)	159(4.2)	202(9.1)	116(12.7)	< 0.001
Dyslipidemia ^b^	15(4.0)	221(5.8)	330(14.9)	212(23.7)	< 0.001
Hypertension ^b^	37(9.7)	697(18.2)	707(31.5)	426(46.6)	< 0.001
Chronic kidney disease ^b^	33(8.6)	199(5.2)	138(6.1)	47(5.2)	0.027
History of medication use					
CVD medications	17(4.4)	210(5.5)	179(7.9)	112(12.2)	< 0.001
Diabetes medications ^b^	0(0.0)	24(0.6)	23(1.0)	9(1.0)	0.085
Hypertension medications ^b^	25(6.5)	442(11.6)	515(23.0)	345(37.7)	< 0.001
Lipid-lowering therapy ^b^	8(2.1)	99(2.6)	141(6.4)	110(12.3)	< 0.001
Blood pressure, mean (SD), mm Hg ^b^					
Systolic	125.4 ± 22.2	126.6 ± 20.0	132.8 ± 20.4	137.2 ± 21.1	< 0.001
Diastolic	71.6 ± 12.1	73.9 ± 11.6	78.5 ± 11.8	81.5 ± 12.1	< 0.001
Metabolic biomarkers ^c^					
Total cholesterol, mean (SD), mg/dL	186.2 ± 35.0	191.1 ± 37.1	197.3 ± 38.1	199.4 ± 39.5	< 0.001
Triglycerides, median (IQR), mg/dL	85.0(65.5,108.9)	95.6(69.0,138.9)	123.9(87.6,180.5)	140.7(99.1,210.6)	< 0.001
High-density lipoprotein, mean (SD), mg/dL	61.0 ± 18.0	53.6 ± 15.6	47.1 ± 13.2	43.3 ± 12.1	< 0.001
Low-density lipoprotein, mean (SD), mg/dL	107.9 ± 30.9	114.6 ± 33.6	120.6 ± 35.2	118.7 ± 36.8	< 0.001
Fasting plasma glucose, mean (SD), mg/dL	104.2 ± 35.5	107.5 ± 34.5	113.7 ± 39.3	116.9 ± 38.5	< 0.001
Estimated glomerular filtration rate, mean (SD), mL/min/1.73 m2	87.0 ± 20.1	88.2 ± 21.3	90.9 ± 21.8	93.9 ± 21.4	< 0.001
High-sensitivity C-reactive protein, median (IQR), mg/L	0.8(0.4,1.9)	0.9(0.5,1.8)	1.1(0.6,2.1)	1.6(0.9,3.2)	< 0.001
Cognitive scores at baseline	14.0 ± 4.5	15.1 ± 4.4	15.9 ± 4.3	16.0 ± 4.5	< 0.001

Abbreviation: SD, standard deviation. IQR, interquartile range (75th quartile minus 25th quartile). ^a^*P* value was based on χ2 or analysis of variance or Mann-Whitney U test where appropriate. ^b^ Missing data: 11 for abdominal obesity, 1 for smoking, 46 for diabetes, 27 for hypertension, 106 for dyslipidemia, 19 for chronic kidney disease, 47 for diabetes medications, 27 for hypertension medications, 110 for lipid-lowering therapy, 80 for systolic blood pressure, and 83 for diastolic blood pressure. ^c^ Measured in a subpopulation of 5582 participants

Compared to their normal-weight counterparts, the obese participants were found to be younger (56.3 ± 7.96 vs. 62.7 ± 9.5, ANOVA *P* <.001), and had a higher prevalence of several chronic conditions. These included CVD (194 [21.2%] vs. 39 [10.2%], chi-square test *P* <.001), diabetes (116 [12.7%] vs. 8 [2.1%], chi-square test *P* <.001), dyslipidemia (212 [23.7%] vs. 15 [4.0%], chi-square test *P* <.001), hypertension (426 [46.6%] vs. 37 [9.7%], chi-square test *P* <.001), and chronic kidney disease (47 [5.2%] vs. 33 [8.6%], chi-square test *P* =.027). Obese patients were also more likely to be taking medication for these conditions, with higher percentages taking medication for CVD (112 [12.2%] vs. 17 [4.4%], chi-square test *P* <.001), hypertension (345 [37.7%] vs. 25 [6.5%], chi-square test *P* <.001), and the lipid-lowering therapy (110 [12.3%] vs. 8 [2.1%], chi-square test *P* <.001) (Table 1). The mean [SD] cognitive function scores at baseline were 14.0 [4.5] scores for underweight, 15.1 [4.4] scores for normal weight, 15.9 [4.3] scores for overweight, and 16.0 [4.5] scores for obese, respectively (*P* <.001).

### Cognitive trajectory modeling

According to the AIC and BIC, four trajectories were identified by the GBTM model, including the high stable (14.9%), the middle stable (46.0%), the middle decline (29.9%), and the low decline groups (9.2%, Table S1). The AvePP values of 4 groups were greater than 0.75, with proportions over 10%. Table S2 displayed the characteristics of these patients categorized by four cognitive function trajectory groups.

### Association between BMI, physical activity with trajectories of cognitive function scores

Table 2 described the association of BMI category and physical activity with 4 trajectories groups of cognitive decline based on multivariable ordered logistic regression. Compared to normal-weight participants, underweight participants were associated with a 26% increase in cognitive trajectory decline risk (adjusted odds ratio [aOR] 1.26, 95% CI, 1.02–1.55), while overweight were associated with a 16% decrease (aOR 0.84, 95% CI, 0.76–0.93), and obese were associated with a 19% decrease (aOR 0.81, 95% CI, 0.71–0.94) after adjusting for age, gender, residence, marital status, educational level, smoking status, drinking status, medical history, and history of medication use. In addition, a 1-SD increase in BMI was associated with a 10% decrease in the cognitive trajectory decline risk (aOR 0.90, 95% CI 0.85–0.95). Table S3 showed that a 1-SD increase in HDL level was associated with a 14.9% increased cognitive trajectory decline risk (aOR 1.149, 95% CI 1.082–1.22). Participants with regular physical activity were independently associated with a lower risk of cognitive trajectory decline (aOR 0.66, 95% CI 0.52–0.84).

**Table 2:** Association between body mass index, abdominal obesity, and physical activity with trajectories of cognitive function scores

Variable	No of patients	Crude OR (95% CI)	Crude P		Adjusted OR (95% CI) ^a^	Adjusted P ^a^		Adjusted OR (95% CI) ^b^	Adjusted P ^b^
**Body mass index, kg/m** ^2^									
Underweight (< 18.5)	384	1.77(1.46,2.15)	< 0.001		1.29(1.05,1.58)	0.014		1.26(1.02,1.55)	0.029
Normal (18.5–23.9)	3,839	1.00(1.00,1.00)	Ref.		1.00(1.00,1.00)	Ref.		1.00(1.00,1.00)	Ref.
Overweight (24–27.9)	2,253	0.71(0.64,0.78)	< 0.001		0.84(0.76,0.93)	0.001		0.84(0.76,0.93)	0.001
Obesity (≥ 28)	916	0.64(0.56,0.73)	< 0.001		0.81(0.71,0.94)	0.004		0.81(0.70,0.94)	0.006
**Per SD**	7,392	0.77(0.74,0.81)	< 0.001		0.89(0.85,0.94)	< 0.001		0.90(0.85,0.95)	< 0.001
**Physical activity**	3,172	0.72(0.57,0.90)	0.004		0.64(0.50,0.81)	< 0.001		0.66(0.52,0.84)	0.001

Abbreviations: SD indicates standard deviation

^a^ Adjusted for age, gender, residence, marital status, educational level, smoking status, and drinking status

^b^ Further adjusted for medical history (CVD, diabetes, hypertension, dyslipidemia, and chronic kidney disease) and history of medication use (CVD medications, hypertension medications, diabetes medications, and lipid-lowering therapy)

### Mediation effect of physical activity

**Fig. 2:**
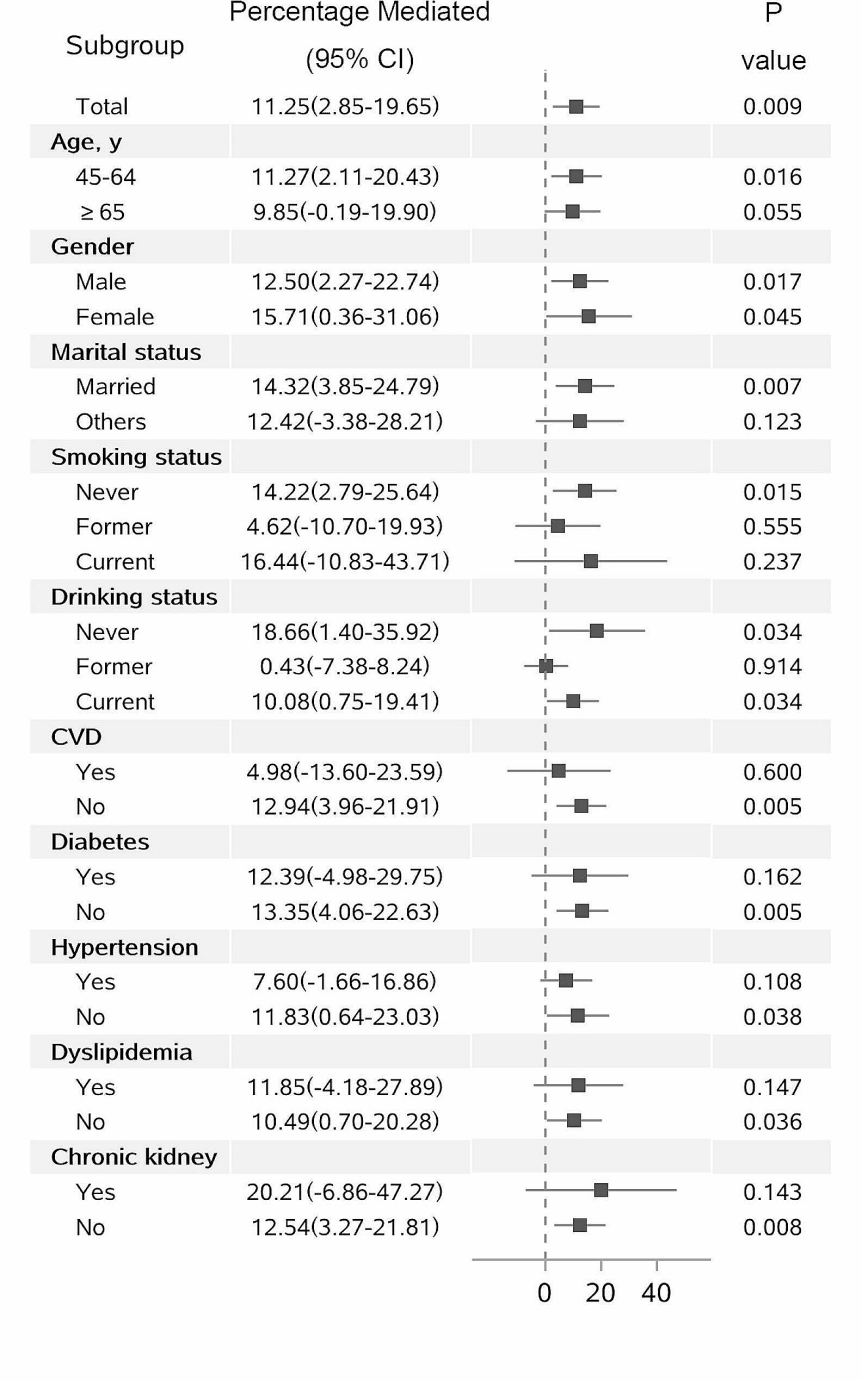
Causal mediation analysis stratified by prespecified subgroup Abbreviation: OR, odds ratio. CVD, cardiovascular disease

Of 3,172 participants with physical activity measures, the total, direct associations, and indirect associations of BMI _per SD_ with trajectories of cognitive decline (decline groups vs. stable groups) mediated by physical activities were presented in Table 3. The result showed that overall physical activity cannot significantly be mediate the relationship between BMI _per SD_ and cognitive decline (adjusted PM 0.14%; 95% CI -1.21-1.50%). After stratified by the intensity of physical activities, vigorous physical activities significantly mediated the association between BMI _per SD_ and cognitive decline (adjusted PM 5.94%; 95% CI 0.29%-11.60%), while moderate and mild physical activities cannot mediate the relation. The indirect association via vigorous physical activities implied that a 1% increase in the risk of cognitive decline (aOR 0.99; 95% CI, 0.97-1.00) would be observed on average. Data across major strata defined by age, gender, marital status, smoking status, drinking status, and history of comorbidities showed comparable results, with the mediated percentage in the range of 0.43–20.21% (Fig. 2).

**Table 3:** Proportion of association of per SD of body mass index with trajectories of cognitive function scores mediated by physical activity

Effect	Model 1			Model 2			Model 3	
	Estimate (95% CI)	P		Estimate (95% CI)	P		Estimate (95% CI)	P
Physical activity (*n* = 3,172)								
Total Effect (TE), Odds Ratio	0.81(0.73,0.89)	< 0.001		0.81(0.73,0.89)	< 0.001		0.82(0.73,0.90)	< 0.001
Natural Direct Effect (NDE), Odds Ratio	0.81(0.73,0.89)	< 0.001		0.81(0.73,0.89)	< 0.001		0.82(0.73,0.90)	< 0.001
Natural Indirect Effect (NIE), Odds Ratio	1.00(1.00,1.00)	0.958		1.00(1.00,1.00)	0.958		1.00(1.00,1.00)	0.834
Percentage Mediated (PM)	-0.03(-1.33,1.26)	0.958		-0.03(-1.25,1.18)	0.958		0.14(-1.21,1.50)	0.834
Vigorous physical activity (*n* = 3,172)								
Total Effect (TE), Odds Ratio	0.81(0.73,0.89)	< 0.001		0.80(0.72,0.88)	< 0.001		0.81(0.72,0.89)	< 0.001
Natural Direct Effect (NDE), Odds Ratio	0.83(0.75,0.91)	< 0.001		0.81(0.73,0.89)	< 0.001		0.82(0.73,0.90)	< 0.001
Natural Indirect Effect (NIE), Odds Ratio	0.98(0.96,0.99)	0.000		0.98(0.97,0.99)	0.002		0.99(0.97,1.00)	0.012
Percentage Mediated (PM)	10.74(3.26,18.22)	0.005		8.06(1.43,14.69)	0.017		5.94(0.29,11.60)	0.040
Moderately energetic physical activity (*n* = 3,169)								
Total Effect (TE), Odds Ratio	0.81(0.73,0.89)	< 0.001		0.81(0.73,0.89)	< 0.001		0.82(0.73,0.90)	< 0.001
Natural Direct Effect (NDE), Odds Ratio	0.81(0.73,0.89)	< 0.001		0.81(0.73,0.89)	< 0.001		0.82(0.73,0.90)	< 0.001
Natural Indirect Effect (NIE), Odds Ratio	1.00(1.00,1.00)	0.898		1.00(1.00,1.00)	0.764		1.00(1.00,1.00)	0.742
Percentage Mediated (PM)	-0.09(-1.54,1.36)	0.898		-0.27(-2.01,1.48)	0.764		-0.25(-1.72,1.23)	0.743
Mildly energetic physical activity (*n* = 3,165)								
Total Effect (TE), Odds Ratio	0.81(0.73,0.89)	< 0.001		0.81(0.73,0.89)	< 0.001		0.82(0.73,0.90)	< 0.001
Natural Direct Effect (NDE), Odds Ratio	0.81(0.73,0.89)	< 0.001		0.81(0.73,0.89)	< 0.001		0.82(0.74,0.90)	< 0.001
Natural Indirect Effect (NIE), Odds Ratio	1.00(0.99,1.00)	0.455		1.00(0.99,1.00)	0.464		1.00(0.99,1.00)	0.365
Percentage Mediated (PM)	0.66(-1.10,2.43)	0.461		0.68(-1.16,2.52)	0.470		0.99(-1.21,3.19)	0.378

Abbreviations: SD indicates standard deviation

*Model 1 was adjusted for age and gender; Model 2 was further adjusted for the interaction of BMI _per SD_ and Physical activity; Model 3 was further adjusted for marital status, medical history (CVD, diabetes, hypertension, dyslipidemia, and chronic kidney disease), and history of medication use (CVD medications, hypertension medications, diabetes medications, and lipid-lowering therapy)

## Discussion

This study examined the cognitive trajectories of a nationally representative sample of 7,392 middle and old-aged adults in China who were followed up for four waves, as well as the relationship between baseline BMI and cognitive trajectories, and the mediating role of physical activity. The results indicated that there were four distinct cognitive trajectories, each associated with different BMI statuses. Additionally, vigorous physical activity partially mediated the association between obesity and cognitive decline, explaining approximately 5.94% of the total effect.

In this study, we found no evidence that overall physical activity played a mediating role, we further investigated the mediating role of three different exercise intensities. Specifically, we explored the mediating effect of vigorous, moderate, and mild regular physical activity on the association between obesity and cognitive decline. Our results indicated that vigorous and regular physical activity partially mediated the association, while moderate or mild regular physical activity did not. We consider that individuals in the middle-aged and elderly cohorts who exhibit the capacity to engage in intense and recurrent physical exertion are frequently characterized by obesity without concurrent organic pathologies. Furthermore, this group tends to be more physically active compared to individuals with normal weight, which in turn slows cognitive decline. On the other hand, individuals with chronic diseases, who may have organic impairments, might mask this mediating effect.

The previous evidence indicated that individuals with obesity may exhibit higher levels of physical activity as a result of their health concerns [13], which corroborated the initial phase of the mediating pathway from obesity to physical activity. Despite previous studies suggesting a mediating role of physical activity, our study represented the first empirical validation of this notion [13]. Furthermore, the second half of our mediation pathway (physical activity to cognitive decline) is corroborated by accumulating evidence that some previous studies showed that physical activity was a protective factor against cognitive decline [23, 24]. Physical activity can help foster healthy lifestyles, improve social relationships, and enhance self-regulation, which are favorable determinants of cognitive function in late adulthood [25].

Our study provided evidence for a statistically significant partial mediating effect of physical activity on the link between obesity and cognitive decline. However, we observed that the magnitude of this effect was relatively small, which implies that there may be other, as yet unknown, factors that are involved in this association. Possible factors that should be taken into account include dietary habits, genetic predispositions, familial support, and social support. Thus, further research is necessary to confirm the presence of these alternative mediators and to investigate their potential influence on the relationship between obesity and cognitive decline, which may provide new opportunities for intervention and prevention strategies. Our results underscore the complexity of the association between obesity and cognitive decline, emphasizing the importance of ongoing research in this field.

The study also observed the “obesity paradox”, and the use of a distinct cognitive function assessment scale and a longer follow-up cohort compared to prior research reinforced this observation. Furthermore, the study population is different from previous studies, and which can effectively improve the extrapolation of the conclusion. To clarify the paradox, we explored the associations between lipids and cognitive trajectory decline. It showed that higher HDL level was associated with an increased cognitive trajectory decline risk. Previous evidence on the relationship between HDL levels and risk of cognitive declines remained controversial [26]. Sultana et al. found a significant association of HDL-C higher than 80 mg/dL with increased accelerated cognitive decline among participants in the Aspirin in Reducing Events in the Elderly (ASPREE) trial [27], while higher HDL-C was reported to be associated with better cognitive function in the Maine Syracuse Study and lower dementia risk in the Japan Public Health Centre-based prospective (JPHC) Study [28, 29]. Furthermore, our study also showed that HDL level was lower in the obesity population, therefore, the metabolic disturbance (higher HDL levels and underweight) was related with higher risk of cognitive trajectory decline.

The current study boasts several notable strengths. Firstly, the study featured a sizeable, nationally representative sample, lending itself to a wide-ranging generalizability of the findings to the general middle-aged and older Chinese population. Secondly, the investigation of longitudinal associations utilizing repeated measures of the study’s variables facilitated a comprehensive comprehension of the direction of the relationship between obesity and cognitive decline. Furthermore, the study evaluated the underlying mechanisms of this association, revealing that the prospective link between obesity and cognitive decline is partially mediated by vigorous physical activity, thereby presenting avenues for the development of health interventions.

Nevertheless, this study has several limitations. Firstly, since the information related to cognitive function scores and covariates was mainly self-reported, recall bias was inevitable. To minimize the bias, we applied the health examination data to identify patients with undiagnosed medical conditions. Secondly, although this prospective study firstly demonstrated the epidemiological associations between obesity, vigorous physical activity, and cognitive trajectory decline, it cannot directly draw a causal relationship. Future Mendelian randomization studies should be applied to establish the potential causal relationships. Thirdly, another potential bias is healthy participant bias because an upward trend of high and middle stable groups was observed in terms of delayed recall. If that was the case, then the observed association between obesity and cognitive trajectory decline possibly could be underestimated. Finally, this study involved only the Chinese population, and the generalizability of our findings should be verified in other ethnic groups to determine whether heterogeneity exists in different ethnicities.

## Conclusion

In summary, our study reveals that vigorous physical exercise accounts for a meager proportion of the correlation between cognitive decline and obesity in middle and old-aged adults. This finding emphasizes the need for further exploration of alternative factors that may serve as mediators in the relationship between obesity and cognitive decline. Additionally, our results underscore the importance of examining the generalizability of our findings to diverse cultural and demographic contexts. Future research endeavors in this field should prioritize investigating these crucial areas.

### Electronic supplementary material

Below is the link to the electronic supplementary material.

**Supplementary Material 1: Table S1.** Permutations of trajectory groups of cognitive function scores and corresponding Bayesian Information Criterion. **Table S2.** The participants' characteristics according to the cognitive function trajectory groups. **Table S3.** Association between lipid metabolites with trajectories of cognitive function. **Figure S1.** Trajectories of the cognitive function score and its five measures (immediate word recall, delayed word recall, orientation, visuo-construction, and attention). The solid lines mean estimated values and the dotted lines display the 95% CIs

## Data Availability

The National School of Development at Peking University provided the data sets in the China Health and Retirement Longitudinal Survey (http://charls.pku.edu.cn/).
