# Supplementary material for: Association between obesity, physical activity, and cognitive decline in Chinese middle and old-aged adults: a mediation analysis
Source: BMC Geriatr. 2024 Jan 11;24:54. doi: 10.1186/s12877-024-04664-4 (PMC10785530; doi:10.1186/s12877-024-04664-4)
Supplement: Supplementary file 1 — Supplementary Material 1: Table S1. Permutations of trajectory groups of cognitive function scores and corresponding Bayesian Information Criterion. Table S2. The participants' characteristics according to the cognitive function trajectory groups. Table S3. Association between lipid metabolites with trajectories of cognitive function. Figure S1. Trajectories of the cognitive function score and its five measures (immediate word recall, delayed word recall, orientation, visuo-construction, and attention). The solid lines mean estimated values and the dotted lines display the 95% CIs [file 12877_2024_4664_MOESM1_ESM.doc]

Table S1. Permutations of trajectory groups of cognitive function scores and corresponding Bayesian Information Criterion

| Number of Groups | Polynomial Order by Group | | | | | AIC | BIC | Proportion | AvePP |
| --- | --- | --- | --- | --- | --- | --- | --- | --- | --- |
|  | G1 | G2 | G3 | G3 | G4 |  |  |  |  |
| 2 | 1 | 1 |  |  |  | -72342 | -72367 | 58.383%/41.617% | 92.760%/90.420% |
| 2 | 2 | 1 |  |  |  | -72343 | -72372 | 58.384%/41.616% | 92.761%/90.419% |
| 2 | 2 | 2 |  |  |  | -72344 | -72367 | 58.383%/41.617% | 92.760%/90.420% |
| 3 | 1 | 1 | 1 |  |  | -71587 | -71624 | 48.908%/28.660%/22.432% | 83.724%/85.885%/87.180% |
| 3 | 2 | 1 | 1 |  |  | -71588 | -71629 | 48.910%/28.659%/22.431% | 83.724%/85.884%/87.182% |
| 3 | 2 | 2 | 1 |  |  | -71589 | -71634 | 48.908%/28.660%/22.432% | 83.724%/85.885%/87.181% |
| 3 | 2 | 2 | 2 |  |  | -71590 | -71639 | 48.907%/28.661%/22.432% | 83.723%/85.886%/87.181% |
| **4** | **1** | **1** | **1** | **1** |  | **-71403** | **-71452** | **43.783%/29.304%/16.718%/10.195%** | **79.851%/76.270%/82.472%/82.286%** |
| 4 | 2 | 1 | 1 | 1 |  | -71405 | -71457 | 43.778%/29.302%/16.719%/10.201% | 79.863%/76.237%/82.475%/82.306% |
| 4 | 2 | 2 | 1 | 1 |  | -71406 | -71462 | 43.781%/29.304%/16.720%/10.195% | 79.851%/76.268%/82.475%/82.288% |
| 4 | 2 | 2 | 2 | 1 |  | -71407 | -71467 | 43.781%/29.304%/16.719%/10.196% | 79.851%/76.268%/82.473%/82.291% |
| 4 | 2 | 2 | 2 | 2 |  | -71408 | -71472 | 43.781%/29.304%/16.720%/10.196% | 79.851%/76.268%/82.474%/82.289% |
| 5 | 1 | 1 | 1 | 1 | 1 | -71409 | -71477 | 33.716%/32.140%/19.983%/8.587%/5.573% | 71.119%/73.926%/72.938%/76.855%/78.722% |

Definition of abbreviations: AIC = Akike Information Criterion; BIC = Bayesian Information Criterion; AvePP = Average posterior probability.

Polynomial order: 1 = linear; 2 = quadratic.

*Group numbers correspond to the order of groups starting from the bottom of the trajectory figure.

Table S2. The participants' characteristics according to the cognitive function trajectory groups

| Characteristic | High stable(n=1103) | Middle stable (n=3399) | Middle decline(n=2214) | Low decline(n=676) | P valve ^a^ |
| --- | --- | --- | --- | --- | --- |
| Age, mean (SD), y | 54.1±7.2 | 56.8±8.0 | 59.9±8.4 | 63.4±9.0 | <0.001 |
| Male | 557(50.5) | 1973(58.0) | 1122(50.7) | 264(39.1) | <0.001 |
| Rural residence | 461(41.8) | 1964(57.8) | 1541(69.6) | 496(73.4) | <0.001 |
| Married | 1008(91.4) | 3000(88.3) | 1851(83.6) | 508(75.1) | <0.001 |
| Educational level |  |  |  |  | <0.001 |
| No formal education | 20(1.8) | 200(5.9) | 562(25.4) | 383(56.7) |  |
| Primary school | 228(20.7) | 1538(45.2) | 1258(56.8) | 257(38.0) |  |
| Middle or high school | 792(71.8) | 1609(47.3) | 392(17.7) | 36(5.3) |  |
| College or above | 63(5.7) | 52(1.5) | 2(0.1) | 0(0.0) |  |
| Smoking status ^b^ |  |  |  |  | <0.001 |
| Never | 689(62.5) | 1861(54.8) | 1238(55.9) | 431(63.9) |  |
| Former | 94(8.5) | 354(10.4) | 218(9.8) | 55(8.1) |  |
| Current | 320(29.0) | 1184(34.8) | 758(34.2) | 189(28.0) |  |
| Drinking status |  |  |  |  | <0.001 |
| Never | 628(56.9) | 1810(53.3) | 1252(56.5) | 416(61.5) |  |
| Former | 65(5.9) | 260(7.6) | 216(9.8) | 65(9.6) |  |
| Current | 410(37.2) | 1329(39.1) | 746(33.7) | 195(28.8) |  |
| History of comorbidities |  |  |  |  |  |
| CVD | 136(12.3) | 502(14.8) | 290(13.1) | 90(13.3) | 0.125 |
| Diabetes ^b^ | 62(5.6) | 238(7.0) | 141(6.4) | 44(6.5) | 0.417 |
| Dyslipidemia ^b^ | 147(13.4) | 385(11.5) | 199(9.1) | 47(7.1) | <0.001 |
| Hypertension ^b^ | 235(21.3) | 872(25.7) | 575(26.1) | 185(27.6) | 0.007 |
| Chronic kidney disease ^b^ | 59(5.4) | 189(5.6) | 126(5.7) | 43(6.4) | 0.832 |
| History of medication use |  |  |  |  |  |
| CVD medications | 52(4.7) | 266(7.8) | 154(7.0) | 46(6.8) | 0.006 |
| Diabetes medications ^b^ | 6(0.5) | 37(1.1) | 10(0.5) | 3(0.4) | 0.026 |
| Hypertension medications ^b^ | 55(5.0) | 173(5.2) | 104(4.8) | 26(3.9) | 0.582 |
| Lipid-lowering therapy ^b^ | 171(15.5) | 630(18.6) | 404(18.3) | 122(18.2) | 0.138 |
| Blood pressure, mean (SD),  mm Hg ^b^ |  |  |  |  |  |
| Systolic | 126.6±17.9 | 129.3±20.2 | 130.7±21.7 | 134.0±23.4 | <0.001 |
| Diastolic | 76.6±11.6 | 76.4±12.1 | 75.7±12.4 | 75.4±12.3 | 0.044 |
| Metabolic biomarkers ^c^ |  |  |  |  |  |
| Total cholesterol, mean (SD), mg/dL | 194.3±39.8 | 192.6±37.1 | 195.2±37.5 | 195.1±38.5 | 0.144 |
| Triglycerides, median (IQR), mg/dL | 109.7(80.5,166.4) | 109.7(76.1,162.0) | 103.5(73.5,152.2) | 100.9(74.3,154.9) | 0.004 |
| High-density lipoprotein, mean (SD), mg/dL | 48.6±14.3 | 50.1±15.3 | 52.0±15.4 | 52.5±15.8 | <0.001 |
| Low-density lipoprotein, mean (SD), mg/dL | 118.4±33.8 | 115.5±34.6 | 117.3±34.8 | 117.0±34.2 | 0.131 |
| Fasting plasma glucose, mean (SD), mg/dL | 109.8±38.4 | 110.3±35.5 | 110.5±36.9 | 112.2±39.9 | 0.682 |
| Estimated glomerular filtration rate, mean (SD), mL/min/1.73 m^2^ | 93.7±21.4 | 88.7±21.8 | 89.1±21.0 | 90.0±21.2 | <0.001 |
| High-sensitivity C-reactive protein, median (IQR), mg/L | 1.0(0.5,1.8) | 1.0(0.6,2.1) | 1.1(0.6,2.2) | 1.0(0.6,2.2) | 0.102 |

Abbreviation: SD, standard deviation. IQR, interquartile range (75th quartile minus 25th quartile). ^a^ P value was based on χ2 or analysis of variance or Mann-Whitney U test where appropriate.

Table S3. Association between lipid metabolites with trajectories of cognitive function

| **Variable**  **Per SD** | **Crude OR**  **(95% CI)** | **Crude P** |  | **Adjusted OR**  **(95% CI) ^a^** | **Adjusted P ^a^** |  | **Adjusted OR (95% CI) ^b^** | **Adjusted P ^b^** |
| --- | --- | --- | --- | --- | --- | --- | --- | --- |
| TG/HDL | 0.972(0.954,0.992) | 0.005 |  | 0.985(0.966,1.004) | 0.114 |  | 0.989(0.969,1.008) | 0.250 |
| TC | 1.062(1.003,1.125) | 0.041 |  | 1.02(0.962,1.082) | 0.506 |  | 1.039(0.979,1.103) | 0.212 |
| TG | 0.942(0.91,0.976) | 0.001 |  | 0.966(0.932,1.001) | 0.055 |  | 0.979(0.943,1.015) | 0.244 |
| HDL | 1.202(1.135,1.273) | <.001 |  | 1.165(1.099,1.235) | <.001 |  | 1.149(1.082,1.22) | <.001 |
| LDL | 1.003(0.947,1.063) | 0.909 |  | 0.944(0.89,1.001) | 0.054 |  | 0.956(0.901,1.015) | 0.142 |

Abbreviations: SD indicates standard deviation; TC, total cholesterol; TG, triglycerides; HDL, high-density lipoprotein, LDL, low-density lipoprotein.

^a^ Adjusted for age, gender, residence, marital status, educational level, smoking status, and drinking status.

^b^ Further adjusted for medical history (CVD, diabetes, hypertension, dyslipidemia, and chronic kidney disease) and history of medication use (CVD medications, hypertension medications, diabetes medications, and lipid-lowering therapy).

| 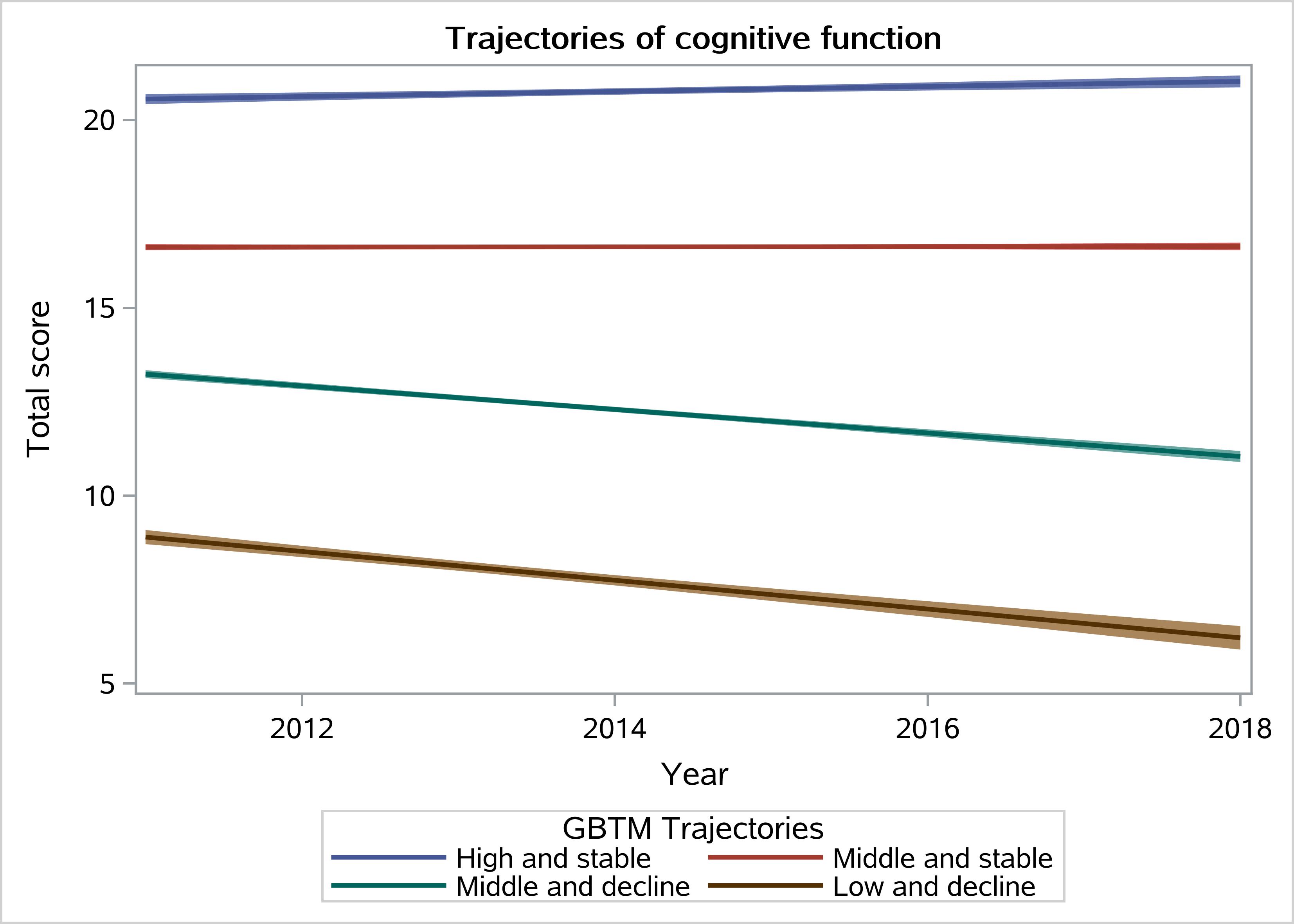 | 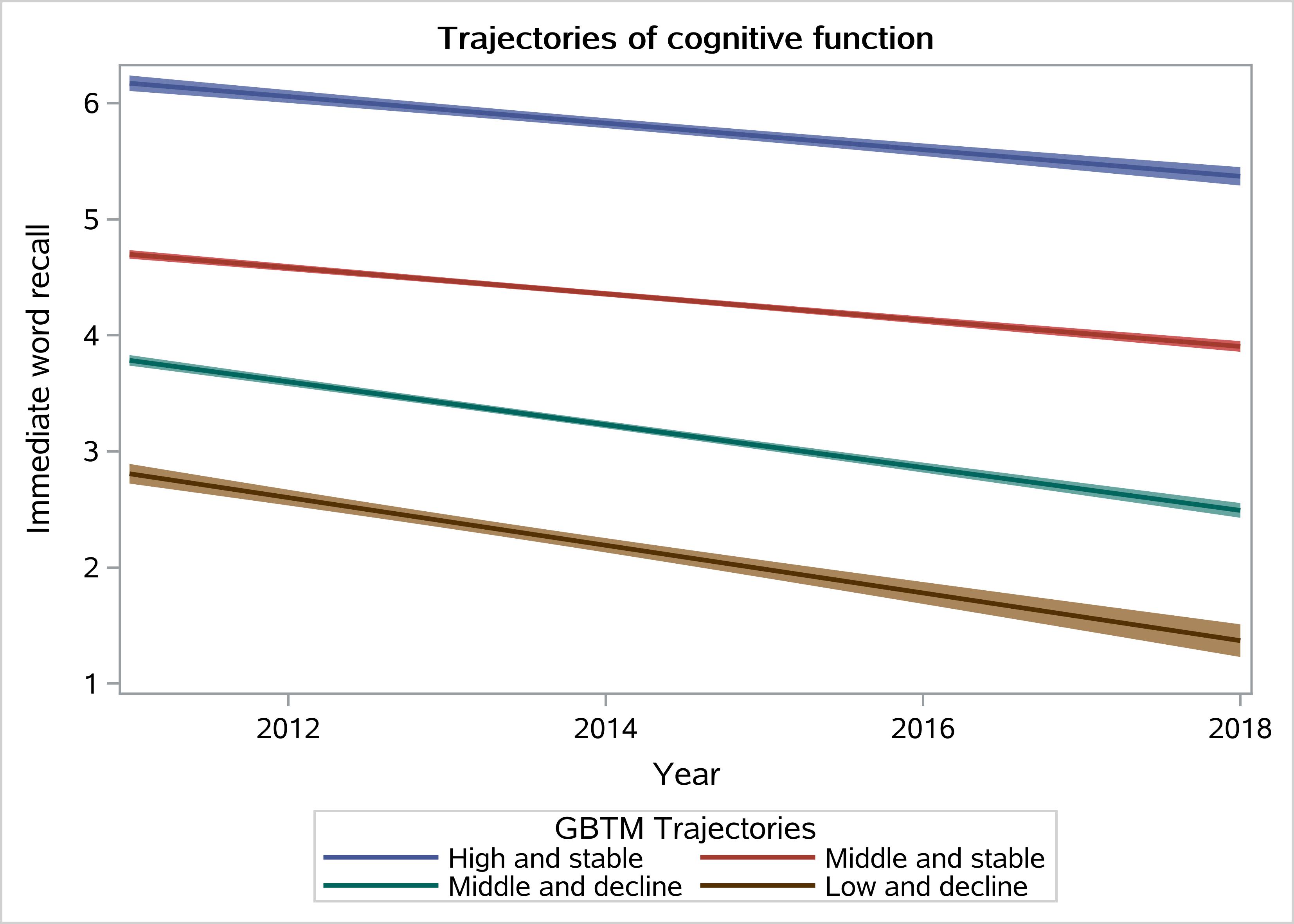 |
| --- | --- |
| 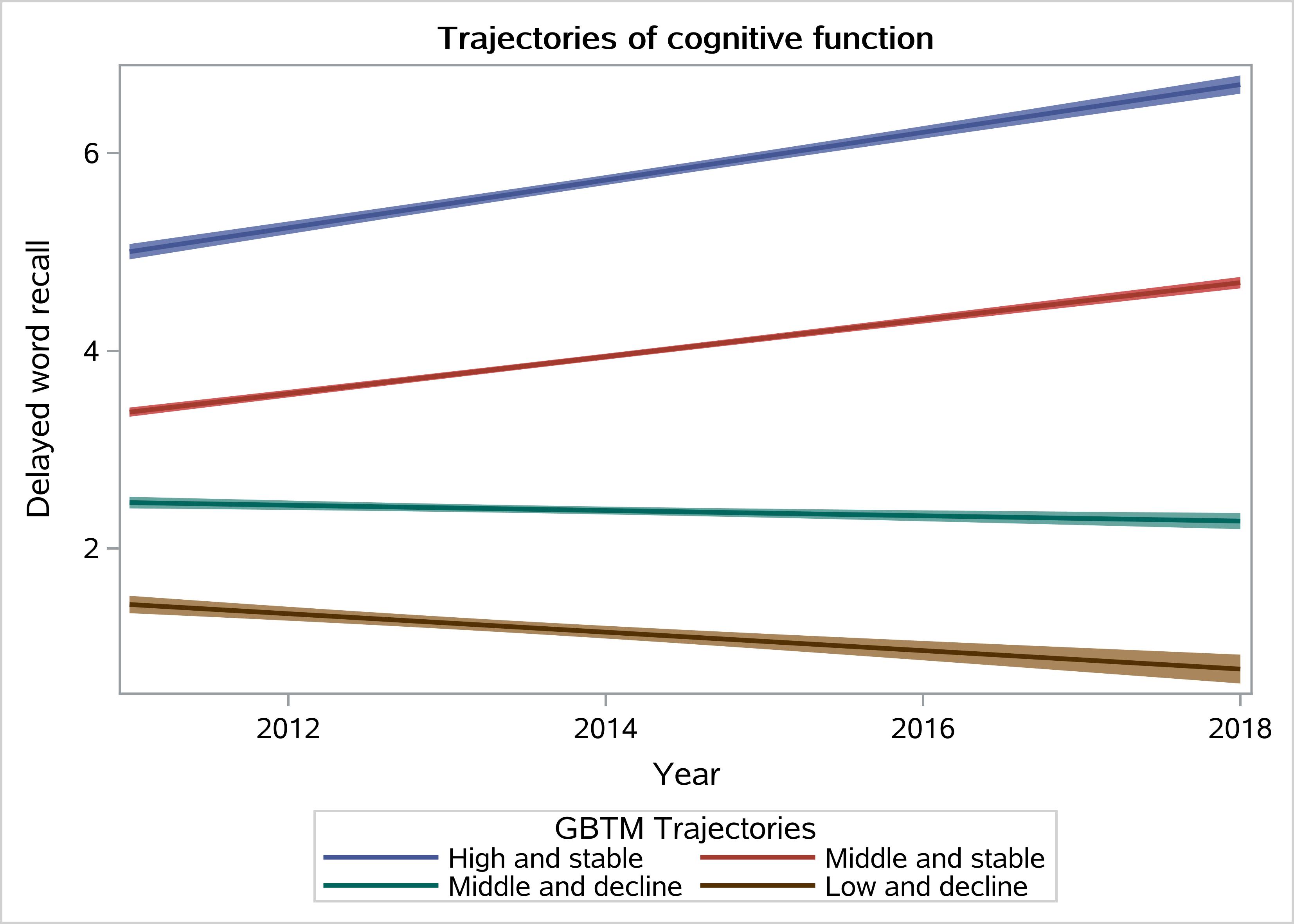 | 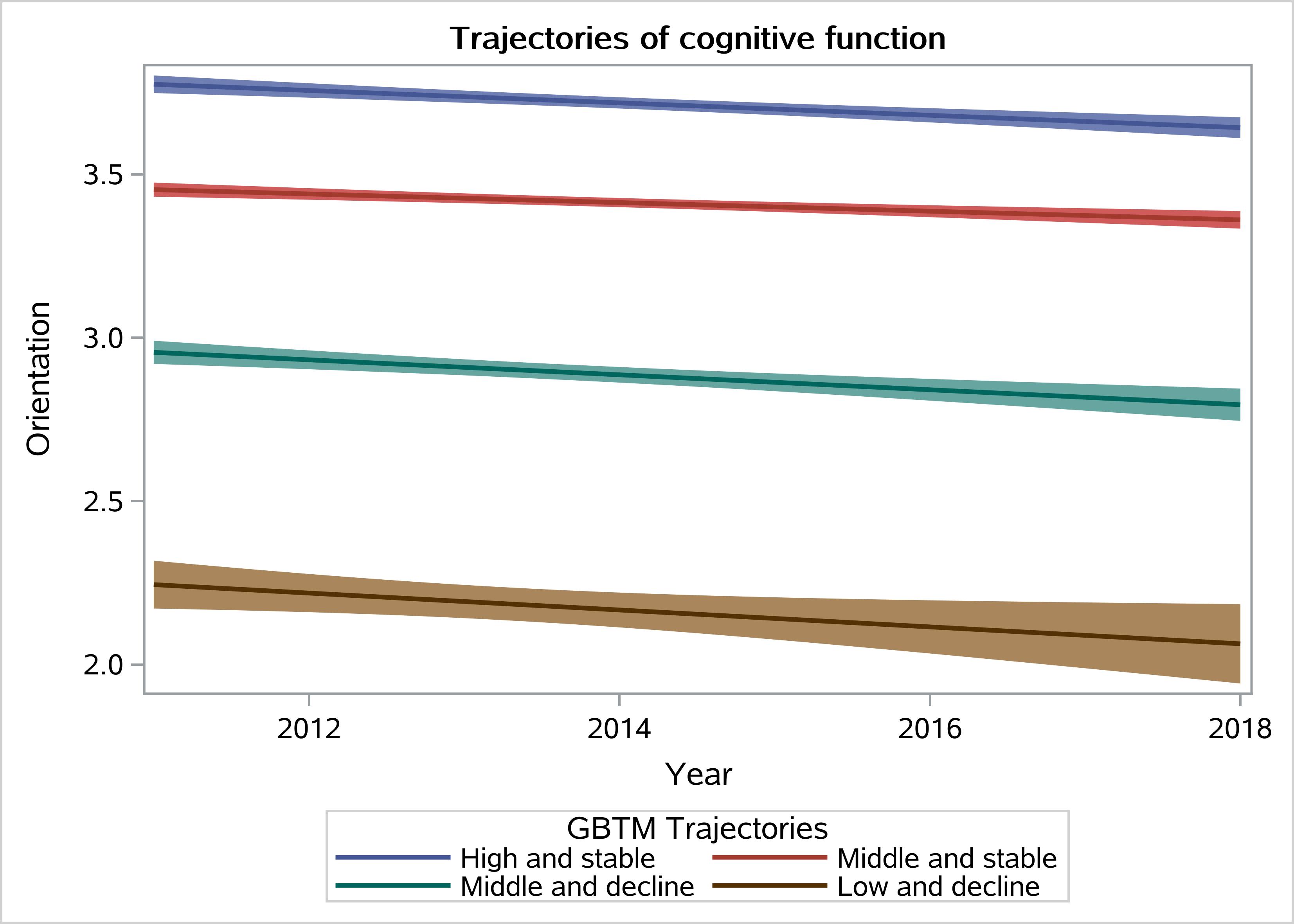 |
| 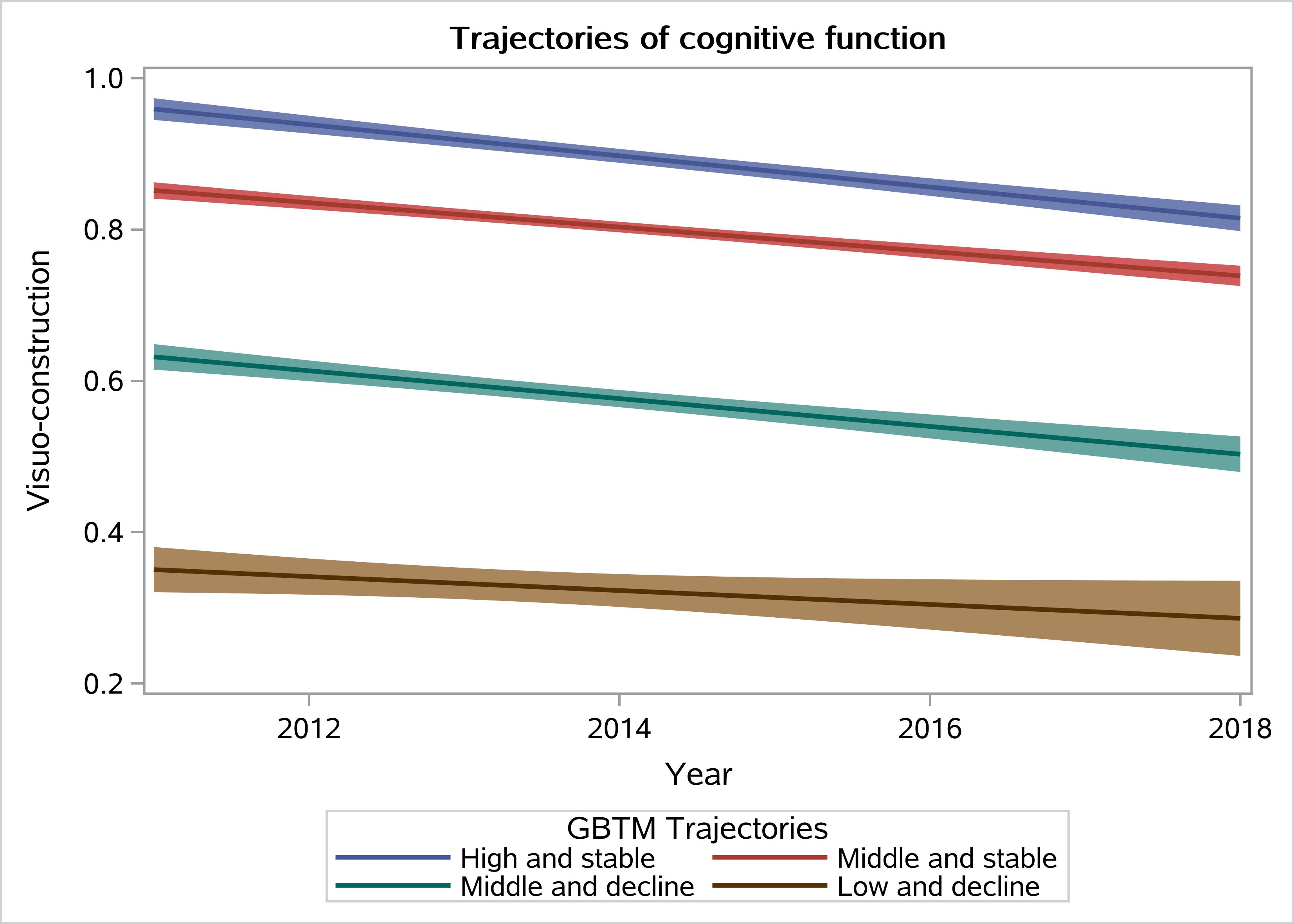 | 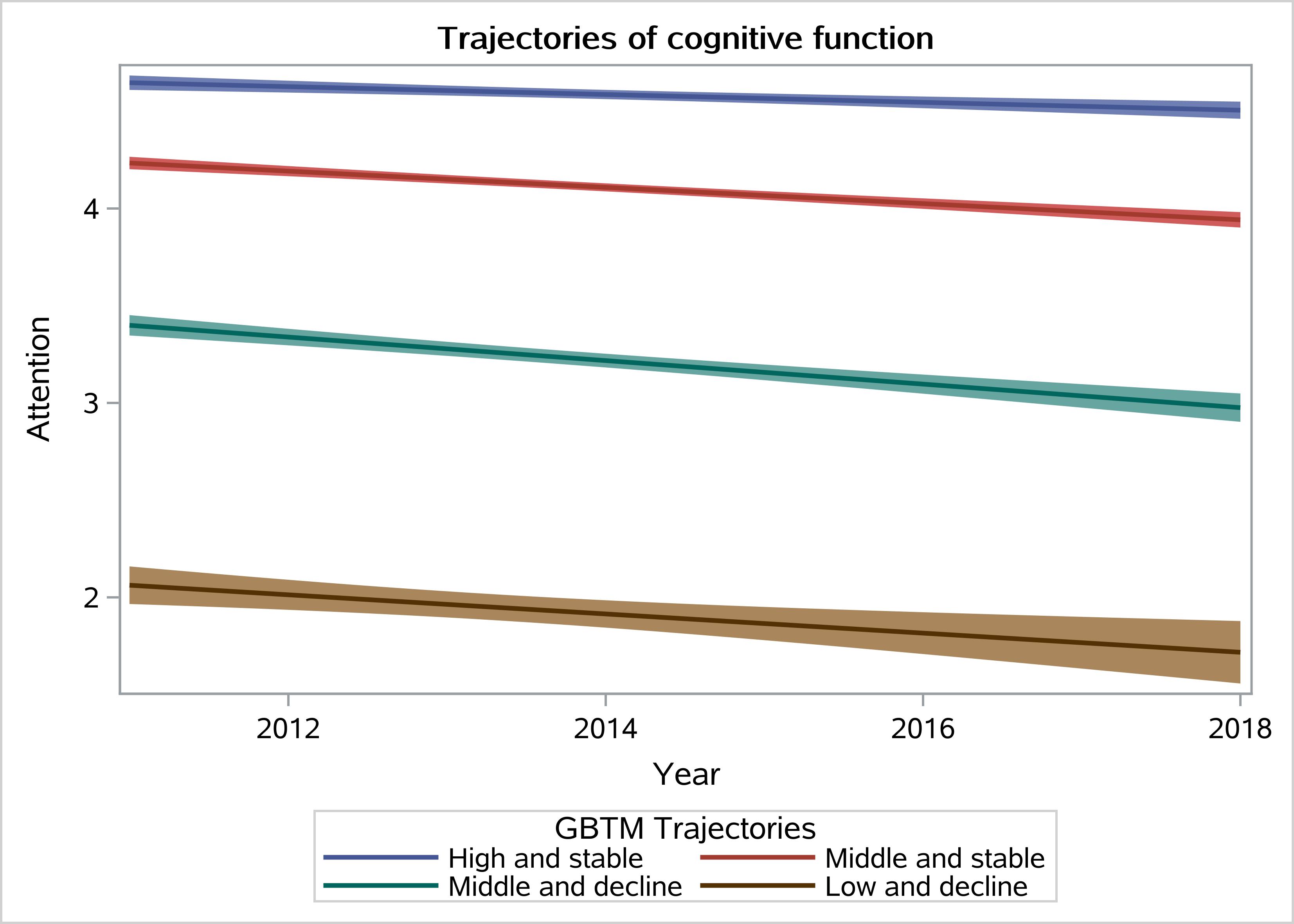 |

Figure S1. Trajectories of the cognitive function score and its five measures (immediate word recall, delayed word recall, orientation, visuo-construction, and attention). The solid lines mean estimated values and the dotted lines display the 95% CIs.
